# A novel glycosidase plate-based assay for the quantification of galactosylation and sialylation on human IgG

**DOI:** 10.1007/s10719-020-09953-9

**Published:** 2020-10-16

**Authors:** Osmond D. Rebello, Richard A. Gardner, Paulina A. Urbanowicz, David N. Bolam, Lucy I. Crouch, David Falck, Daniel I. R. Spencer

**Affiliations:** 1 Ludger Ltd, Culham Science Centre, Abingdon, UK; 2 Center for Proteomics and Metabolomics, Leiden University Medical Center, Leiden, Netherlands; 3 Biosciences Institute, Faculty of Medical Sciences, Newcastle University, Newcastle upon Tyne, UK; 4 Institute of Microbiology and Infection, School of Biosciences, University of Birmingham, Birmingham, B15 2TT UK

**Keywords:** Glycomics, Plate assay, HPLC-FLD-MS, Glycosidase, Glycans, Galactosylation, Sialylation, Antibodies

## Abstract

Changes in human IgG galactosylation and sialylation have been associated with several inflammatory diseases which are a major burden on the health care system. A large body of work on well-established glycomic and glycopeptidomic assays has repeatedly demonstrated inflammation-induced changes in IgG glycosylation. However, these assays are usually based on specialized analytical instrumentation which could be considered a technical barrier for uptake by some laboratories. Hence there is a growing demand for simple biochemical assays for analyzing these glycosylation changes. We have addressed this need by introducing a novel glycosidase plate-based assay for the absolute quantification of galactosylation and sialylation on IgG. IgG glycoproteins are treated with specific exoglycosidases to release the galactose and/or sialic acid residues. The released galactose monosaccharides are subsequently used in an enzymatic redox reaction that produces a fluorescence signal that is quantitative for the amount of galactosylation and, in-turn, sialylation on IgG. The glycosidase plate-based assay has the potential to be a simple, initial screening assay or an alternative assay to the usage of high-end analytical platforms such as HILIC-FLD-MSn when considering the analysis of galactosylation and sialylation on IgG. We have demonstrated this by comparing our assay to an industrial established HILIC-FLD-MSn glycomic analysis of 15 patient samples and obtained a Pearson’s r correlation coefficient of 0.8208 between the two methods.

**Electronic supplementary material:**

The online version of this article (10.1007/s10719-020-09953-9) contains supplementary material, which is available to authorized users.

## Introduction

Changes in IgG glycosylation have been associated with certain inflammatory diseases such as rheumatoid arthritis [1, 2], systemic lupus erythematosus [3], type 2 diabetes [4] and inflammatory bowel disease (IBD) [5]. During an inflammatory state, a loss of systemic and/or cellular homeostasis may trigger a change in the glycosylation of IgG molecules [6, 7]. These changes have been shown to alter the IgG glycoprotein interactions to FcγR receptors among other proteins, and thus tuning the immunoregulatory responses [8–10]. For example, the afucosylation of Fc glycans induces a pro-inflammatory response through interaction with FcγRIIIA receptor [11], whilst their hyper-galactosylation further strengthens this interaction [12, 13]. Masking exposed galactose epitopes on Fc glycans by increased sialylation could induce an anti-inflammatory response through interactions with the immune suppressing DC-SIGN receptor [14, 15]. However, increased agalactosylation of these glycans counteracts this immune suppressive ability by directly reducing the abundance of sialylated epitopes. Additionally, these agalactosylated glycans further induce a pro-inflammatory response via activation of the complement system through interaction with the mannose binding proteins [16]. Although it is clear that traits of fucosylation, galactosylation and sialylation have their own important function in an immunoregulatory response, we are especially interested in galactosylation and sialylation since they are widely known for having mutually opposing immunoregulatory effects, whilst being dependent on each other for their exposure at the non-reducing termini of the glycans. Hence, we focus here on the quantification of galactosylation and sialylation on IgG as they are indicative of the pro-inflammatory and anti-inflammatory states of the patient, respectively.

Glycomic assays are valuable analytical tools to draw associations between an *N*-glycome and the disease state of patients for diagnostic and stratification purposes. These assays are often complicated by the composition and linkage diversity of the glycans and hence they rely heavily on high-end analytical instrumentation [17–19]. The IgG glycome especially, has been widely studied using assays based on the modern iterations of liquid chromatography (LC) with fluorescence detection (FLD) [1, 20, 21], capillary gel electrophoresis (CGE) with laser-induced fluorescence (LIF) detection [22, 23] and matrix assisted laser desorption mass spectrometry (MALDI-MS) [24, 25]. These analytical platforms have demonstrated a multitude of times the ability to perform detailed and high-throughput analyses of IgG glycosylation, and have deeply progressed our understanding of the IgG glycome. It is due to this work that the IgG glycome is now widely regarded as a simple glycome consisting of only 37 *N*-glycans over the 4 subclasses of IgG [24]. These glycans are predominantly biantennary complex type glycans which are mostly core fucosylated [26] while a smaller proportion have bisecting GlcNAc motifs (~13% abundance) [24]. Furthermore, the sialylation and galactosylation are exclusively α(2–6) and β(1–4) linked, respectively. Thus rather than analyzing all glycan variants by using any of the aforementioned analytical platforms, the sole quantification of the inflammatory relevant galactose and sialic acid epitopes could be potentially valuable in drawing associations between a patient’s inflammatory state and the immune modulation by IgG glycosylation. Our research focused on developing such a method as a simple microtitre plate-based biochemical assay that could bypass the use of the high-end analytical platforms. This is an advantage as there is often a frequent high barrier to entry into the use of such analytical platforms as they usually require a substantial amount of time, skill and investment to establish in a laboratory. Furthermore, the commonly used industry platform, LC-FLD, often requires extensive measurement time and complex data processing.

Several sandwich immuno/lectin-based biochemical assays have been previously investigated in the quantification of simple glycan epitopes such as sialylation, galactosylation and fucosylation, to more complex epitopes such as the Lewis epitopes [27–31]. These assays often work on the principle of immuno capturing specific glycoproteins from a sample such as serum or plasma onto a microtiter plate, microarray slide or microfluidic cell, followed by detecting their glycosylation using probing lectins or antibodies that bind to specific glycan epitopes. Techniques that are based on microarray slide or microfluidic cells often have the advantage of being able to analyze multiple glycan epitopes on multiple specific glycoproteins in a potentially high-throughput manner and using low amount (< 10 μL) of serum sample [27, 29, 31]. However, when based on microtitre plates, this method may require large amounts of serum sample dependent on the abundance of the glycoprotein of interest [30]. Assays based on a microarray slides also require substantial knowledge and/or investment in conjugation chemistry, microarray printers and readers. In general, the accuracy of these techniques is highly dependent on the specificity of the antibodies that are used for capturing the glycoproteins of interest and the lectin or antibodies that are used for detecting their glycan epitopes. The sensitivity of the assay also depends on the binding affinity and/or steric hindrances of the detection lectin or antibody. Also, the necessity to chemically block the glycans of the capturing antibodies, so as to reduce background, may result in a loss of binding affinity probably due to the influences on their three-dimensional structure [32, 33].

Building on the need to achieve a simple and easily adoptable assay for glycan epitope quantification, we have developed a novel glycosidase plate-based assay for the quantification of galactosylation and sialylation on IgG glycoproteins. We aimed at overcoming some of the disadvantages of typical sandwich immuno/lectin-based assays such as adoptability and infrastructure investment, by designing our assay for a potential kit-like format that required a microtitre plate reader as the only instrumentation. IgG captured from human plasma was treated with exoglycosidases, and the released β,D-Galactose monosaccharides were used in a coupled enzymatic redox reaction to produce a fluorescence signal which is directly proportional to the amount of released galactose residues. Furthermore, we made a comparison between the assay and an industry established hydrophilic interaction liquid chromatography (HILIC)-FLD-MSn method for the quantification of a galactosylation trait on IgG from 15 serum samples of patients suspected of having IBD.

## Materials and methods

### Reagents and samples

Monosodium dihydrogen phosphate, disodium hydrogen phosphate, formic acid, resazurin, lyophilized diaphorase (Lipoamide Dehydrogenase from *Clostridium kluyveri*) and Protein G beads (Protein G Sepharose®, Fast Flow) were purchased from Sigma Aldrich (UK). Nicotinamide adenine dinucleotide (NAD^+^) was purchased from Roche (Germany). Phosphate buffered saline (PBS) powder, pH 7.4 was purchased from Thermo Fisher Scientific (UK). Galactose monosaccharide standard (400 μg/mL), the buffer pH 8.5 that is used in the preparation of the reagent mix, and the solution of galactose dehydrogenase and mutarotase that is used in the preparation of the redox enzyme mix was obtained from a galactose quantification kit (K-Arga) which was purchased from Megazyme (Ireland). IMAC purified recombinant forms of the β(1–4) specific GH2 galactosidase Bt0461 [34] and α(2–3/6/8) specific GH33 sialidase Bt0455 [35] were provided by Newcastle University. The protein concentrations were determined to be 420 μM and 23 μM, respectively, by a protein quantification kit (Pierce BCA protein assay kit, Thermo Fisher Scientific, UK).

Sodium phosphate buffer solution (SPBS; 250 mM), pH 6 was prepared by dissolving 30.8 g of monosodium dihydrogen phosphate and 4.95 g of disodium hydrogen phosphate in a liter of deionized water and then filtering the solution through a 0.45 μm filter.

IgG glycoprotein standard from human serum was purchased from Sigma Aldrich (UK). The protein concentration was determined to be 6.13 mg/mL (~41 μM) by a protein quantification kit (Pierce BCA protein assay kit) and which was in accordance with the manufacturer. Human plasma standard (containing 4% trisodium citrate as anticoagulant) was purchased from Sigma Aldrich (UK). Human patient serum samples (*n* = 15)were obtained from the IBD-BIOM cohort and which were collected as part of a biobank as was previously described [36]. The patient blood were collected in 3.5 ml vacuette plastic SST II Advance tube with gel separator, clot activator, and BD Hemograd closure (BD, no 367956).The serum obtained from these preparations were used in the experiments of this paper.

### Glycosidase plate-based assay

Briefly, the assay involves affinity purification of IgG glycoprotein from human plasma and quantifying protein amount by measuring absorbance at 280 nm. These IgG glycoproteins are treated with exoglycosidases to release galactose and/or sialic acid residues from the glycans. The released galactose monosaccharides are then subjected to an enzymatic redox reaction to produce a fluorescence output that is measured in a microplate reader.

#### Affinity purification of IgG glycoproteins from human plasma

IgG purification is performed in a 96 well plate format using protein G conjugated sepharose beads. 5 μL of protein G bead resin is transferred to each well of a 96 well plate (LC-PROC-96, Ludger, UK). The plate is fitted onto a vacuum manifold (LC-VAC-MANIFOLD-KIT, Ludger) and the beads are washed three times with 200 μL PBS by applying a vacuum of between 5 and 10 mbars. The plate is removed from the manifold, and 100 μL PBS is added to the wells containing the protein G beads followed by 10 μL of plasma samples. The plate is sealed and is incubated for 60 min at room temperature on an orbital plate shaker at low speed. After incubation, the plate is once again fitted onto a vacuum manifold and the solution in the wells drained away. The protein G beads that have bound the IgG molecules are washed three times with 200 μL PBS, followed by three washes with 200 μL deionized water to wash away residual PBS. The plate is once again removed from the manifold, and the bottom is gently tapped dry on a paper towel. To elute the IgG from the protein G resin, the plate is stacked onto a 96 well collection plate (1.2 mL 96 Deepwell plate, 4titude, UK) and 30 μL of an aqueous solution of 10 mM formic acid is added to the wells containing the resin. The top plate is covered with a lid and incubated for 15 min at room temperature on a plate shaker at low speed. After incubation, the stack plate setup is centrifuged at 1000 rpm for 5 min. The elution procedure is repeated a second time. The eluted IgG samples from the collection plate are then transferred to a conical bottom 96 well PCR plate (Framestar 96 non skirted, 4titude).

#### Protein quantification and exoglycosidase treatment

Each IgG sample was subjected to three treatment conditions which are 1) galactosidase treatment for quantification of terminal galactosylation, 2) combined galactosidase and sialidase treatment for quantification of total galactosylation and 3) exoglycosidase untreated negative control for protein quantification and for sample fluorescence background and interference subtraction. All the following steps for assay preparation were performed on a robotic platform (Microlab starlet, Hamilton, Germany). The IgG sample plate and an empty 384 well plate (Ultravision plate 384, 4titude) were placed on the deck of the robotic platform along with 2 mL tubes (Sarstedt, UK) containing the galactosidase mix (2 μM galactosidase Bt0461 in 125 mM SPBS, pH 6), galactosidase and sialidase mix (2 μM galactosidase Bt0461 and 5 μM sialidase Bt0455 in 125 mM SPBS, pH 6) and 125 μM SPBS, pH 6. For each sample, 20 μL of the respective mixes were transferred to separate wells in the 384 well plate (Supplementary fig. S1). Additionally, 20 μL of the 125 mM SPBS buffer was transferred to the blocks of wells of the galactose monosaccharide standards and of the IgG glycoprotein standards (Supplementary fig. S1). 10 μL of PBS was transferred to the IgG samples to prevent sticking of protein to the plasticware. 15 μL of these IgG samples were transferred each as three sub-samples to the 384 well plate containing the corresponding treatment mixes (Supplementary fig. S1). After this step, the IgG sample plate on the deck was swapped for another plate containing the IgG glycoprotein standards of known amounts (in 10 mM formic acid) for the preparation of the IgG glycoprotein absorbance standard curve. Again, 10 μL of PBS was added to these standards and 15 μL were transferred each to their respective block on the 384 well plate (Supplementary fig. S1). The final amounts of the IgG glycoprotein standards making up the standard curve in the 384 well plate were 30.7 μg, 61.3 μg, 92 μg, 122.6 μg and 184 μg.

The 384 well plate was removed from the deck and centrifuged for 2 min at 1000 g. The plate was placed on an orbital plate shaker for 3–5 min before centrifuging again. Protein amounts of the IgG samples were determined by measuring the absorbance at 280 nm for the exoglycosidase untreated sub-samples and equating it to the absorbance standard curve prepared from the IgG glycoprotein standards of known amounts. The absorbance measurements at 280 nm were taken on a microplate reader (Enspire 2300, Perkin Elmer Enspire, USA). Once the plate was inserted into the reader, it was shaken in an orbital motion for 60 s before the measurements were started, so as to ensure equilibration of its temperature to the chamber which was 21 °C. The measurements were taken at a height of 2 mm. 10 flashes were taken for each well, of which 5 were integrated. The plate was measured three consecutive times and the measurements were averaged.

After measuring the absorbance, the plate was heat sealed (Peel heat seal, 4titude) and incubated overnight at 37 °C.

#### Enzymatic redox reaction and fluorometric measurement of assay

After the overnight exoglycosidase treatment, the samples were subjected to an enzymatic redox reaction which produces the detection mechanism of the assay. All the following steps were performed on the robotic platform. The 384 well plate containing the exoglycosidase treated samples, a fresh conical bottom 96 well PCR plate, and 2 mL and 0.5 mL tubes (Sarstedt) containing the reagent mix [5 μL of pH 8.5 buffer (K-Arga kit, Megazyme), 4 μL of 1 mM resazurin, 2.6 μL of 25 mM NAD^+^ and 23.4 μL of deionized water] and the redox enzyme mix [4 μL of 4 U/mL diaphorase and 1 μL solution of galactose dehydrogenase and mutarotase (K-Arga kit, Megazyme)] respectively, were placed on the deck of the robotic platform. The PCR plate was used for the preparation of a dilution series of galactose monosaccharide in water from a 400 μg/mL galactose stock solution (K-Arga kit, Megazyme, Ireland). This dilution series were prepared in triplicates and 15 μL of each was transferred to the 384 well plate. The final amounts of galactose monosaccharide standards in the 384 well plate were 8.3 pmols, 41.6 pmols, 83.3 pmols, 166.6 pmols, 333.2 pmols, 499.7 pmols and 666.3 pmols. Next, 35 μL of the reagent mix were transferred to the wells containing the samples and the diluted galactose monosaccharide standards. This was followed by transferring 5 μL of the redox enzyme mix to the same wells. The 384 well plate was sealed (qPCR Seal, 4titude) and centrifuged for 2 min at 1000 rpm. The plate was then placed on an orbital plate shaker for 5 min before centrifuging again and incubating in the dark at room temperature for 3 h. After 3 h, 25 μL of PBS was added to the wells of the plate. The plate was placed on an orbital plate shaker for 5 min before centrifuging for 2 min at 1000 rpm.

Fluorescence measurements were taken on the fluorescence microplate reader (Enspire 2300, Perkin Elmer Enspire, USA). The resorfurin that was produced in the redox reaction was quantified by measuring its fluorescence (with an excitation wavelength (λex) = 571 nm and an emission wavelength (λem) = 586 nm). The excitation illumination was from above the plate and the measurements were taken at a height of 10 mm. 100 flashes were taken for each well and were all integrated. The plate was measured three consecutive times and the measurements were averaged.

A standard curve was prepared from the fluorescence measurements of the galactose monosaccharide standard (K-Arga, Megayzme) solutions of known amounts. For each sample, the fluorescence measurement from the exoglycosidase untreated sub-sample was subtracted from the sub-samples treated with galactosidase and the combination of galactosidase and sialidase, respectively. This was done to exclude fluorescence background or interferences coming from the sample itself. The amount of galactosylation in the samples was calculated by equating their fluorescence measurements to the galactose standard curve.

#### Method optimization and assay performance

For details pertaining to assay development and optimization please see section “Supplementary Methods”. The linear range of quantification of the assay for IgG glycoproteins was determined with human IgG glycoprotein standard (Sigma, UK). These standards were dried down using a vacuum centrifuge (Thermo-Savant, UK) and then reconstituted in PBS to make solutions of 30.7 μg/10 μL, 61.3 μg/10 μL, 92 μg/10 μL, 122.6 μg/10 μL and 245.2 μg/10 μL.

For demonstrating the assays ability to remove all possible galactose and/or sialic acid residues from the *N*-glycans on the intact IgG glycoproteins, IgG samples purified from human plasma were treated with the exoglycosidase regime as according to the workflow of the assay. However, after the overnight exoglycosidase treatment, these sub-samples were collected for HILIC-FLD-MSn analysis (see subsection 2.4).

The intermediate precision of the assay for sample preparation and measurement was performed in three independent experiments on three different days within a period of three weeks. For each of these experiments, the assay was performed with 16 replicates of IgG purified from a pooled human plasma (Sigma Aldrich).

### Hydrophilic interaction liquid chromatography analysis of IgG *N*-glycans

PNGaseF released *N*-glycans from human IgG were used for procainamide labelling and analysis on a HILIC-FLD-MSn platform as previously described [37]. IgG was affinity purified from human plasma/serum as described in section 2.2.1. 25 μL of the purified IgG samples were dried down in the 96 well PCR plate using a vacuumed centrifuge (manufacturer) before resuspending them in 9 μL water. The *N*-glycans were released using a PNGaseF kit (LZ-rPNGaseF-kit, Ludger). Briefly, the IgG glycoproteins were denatured by adding 1 μL of 10x denaturation buffer to the samples, heat sealing (Pierce heat seal, 4titude) the plate and incubating at 95 °C for 10 min. After heat denaturation, the plate was allowed to cool to room temperature before adding 10 μL of a PNGaseF mix [1 μL PNGaseF, 2 μL 10x reaction buffer, 2 μL 10x NP-40 and 5 μL water]. The sample plate was heat sealed (Pierce heat seal, 4titude) before incubating it overnight at 37 °C.

Following the overnight incubation, the reducing ends of the released glycans were deaminated to aldoses by acidifying the samples in 1% formic acid (Sigma) at room temperature for 50–60 min. These acidified samples were then filtered through a protein binding plate (LC-PBM-96, Ludger). The wells of the plate were washed twice with 100 μL water. The washes collected along with the filtrate were dried down in a vacuum centrifuge. These *N*-glycans samples were labelled by reductive amination in 10 μL of water and 10 μL of procainamide labelling solution (LT-KPROC-24 containing NaCNBH_3_, Ludger), and incubated for 60 min at 65 °C. A HILIC-type clean-up plate (LC-PROC-96, Ludger) was used to remove unreacted procainamide dye. Procainamide labelled *N*-glycans were eluted twice in 100 μL water. These purified glycan samples were dried down in vacuum centrifuge and resuspended in 100 μL water.

Procainamide labelled samples were analyzed by HILIC-FLD-MSn. 12.5 μL of each sample was mixed with 37.5 μL acetonitrile, and 20 μL of this solution was injected into an ACQUITY BEH Glycan column (1.7 μm, 2.1 × 150 mm Waters Inc., USA) at 60 °C on a Dionex Ultimate 3000 UHPLC instrument (Thermo, UK) with a fluorescence detector (λex = 310 λem = 370 nm) coupled in-line to an Amazon speed ETD (Bruker Daltonics, Bremen, Germany). The UHPLC gradient conditions were as follows: (solvent A – 50 mM ammonium formate, pH 4.4; solvent B – acetonitrile) 0 to 53.5 min, 76 to 51% B, 0.4 mL/min; 53.5 to 55.5 min, 51% to 0% B, 0.4 mL/min to 0.2 mL/min; 55.5 to 57.5 min, 0% B at a flow rate of 0.2 mL/min; 57.5 to 59.5 min, 0 to 76% B, 0.2 mL/min; 59.5 to 65.5 min, 76% B, 0.2 mL/min; 65.5 to 66.5 min, 76% B, 0.2 mL/min to 0.4 mL/min; 66.5 to 70.0 min, 76% B, 0.4 mL/min. The Amazon Speed settings used were as follows: source temperature, 250 °C; gas flow, 10 L/min; capillary voltage, 4500 V; ICC target, 200,000; Max. accu. Time (Maximum Accumulation Time), 50.00 ms; rolling average, 2; number of precursor ions selected, 3; release after 0.2 min; positive ion mode; scan mode, enhanced resolution; mass range scanned, 600 to 2000; target mass, 900.

## Results and discussion

### Workflow and mechanism of assay

**Fig. 1:**
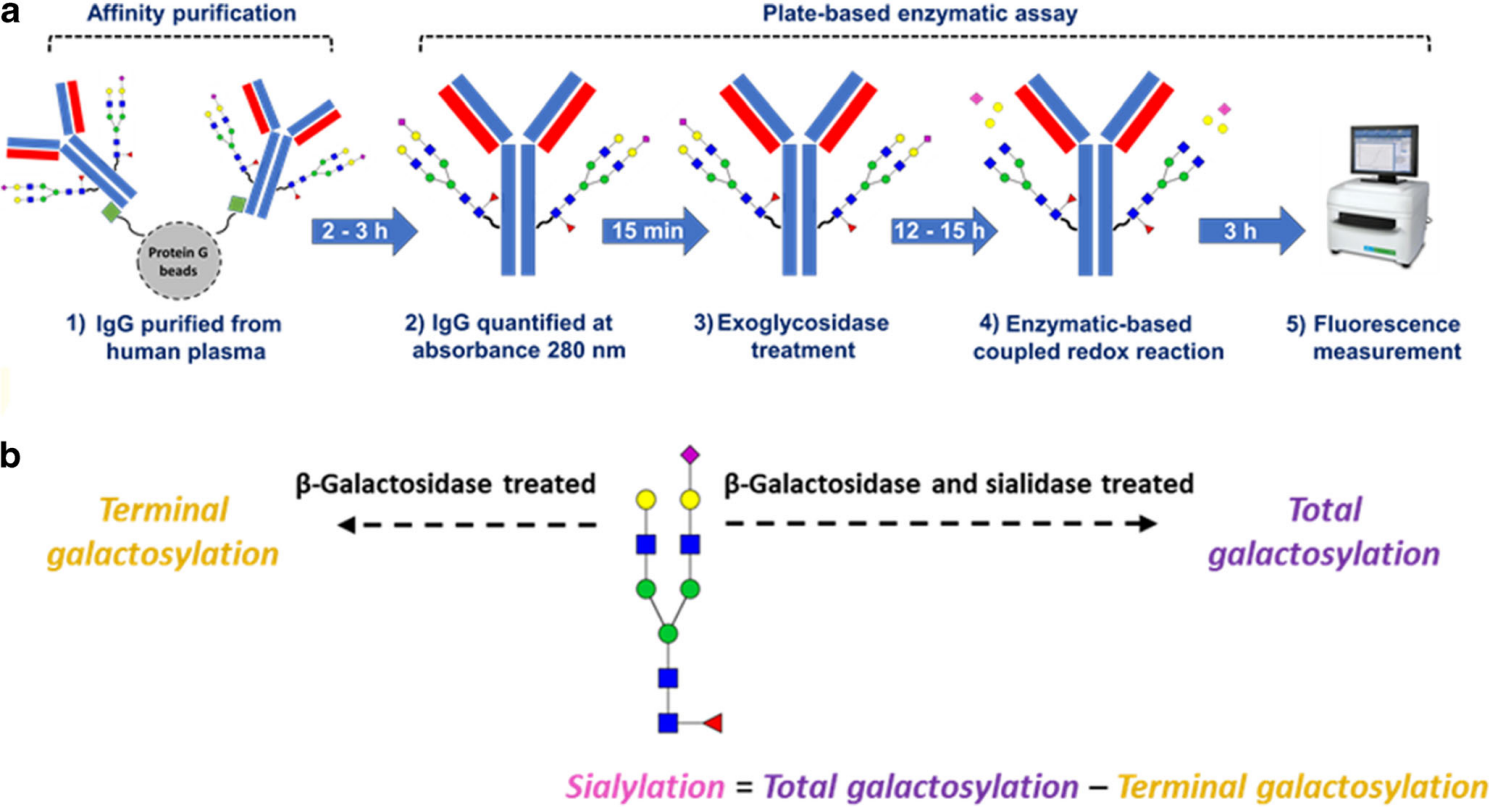
(**a**) Workflow of the glycosidase plate-based assay for the quantification of galactosylation and sialylation on affinity purified IgG from human plasma. (**b**) Schematic description of exoglycosidase treatment regime for the quantification of total or terminal galactosylation. The difference between both quantitated galactosylation types is equivalent to the sialylation on the glycoproteins not typically exhibiting polysialylation. [Green squares: protein G; Blue square: *N*-acetylglucosamine, green circle: mannose, yellow circle: galactose, red triangle: fucose, pink diamond: *N*-acetylneuraminic acid]

**Fig. 2:**
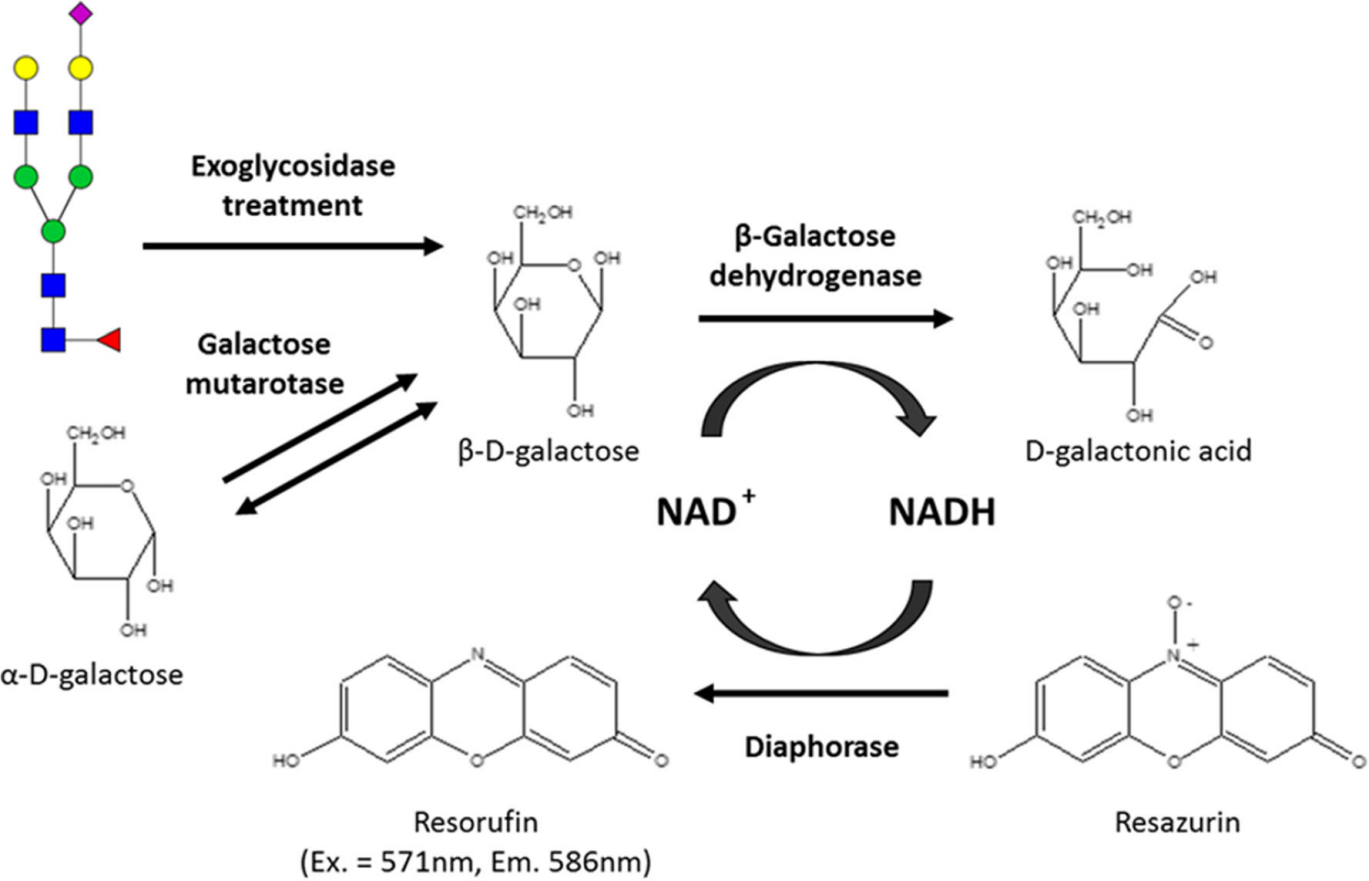
Schematic of the enzymatic redox reaction resulting in fluorescent compound resorufin that forms the detection mechanism of the glycosidase plate-based assay. Resorufin measured at excitation (Ex.) wavelength of 571 nm and emission (Em.) wavelength of 586 nm. [NAD^+^: Nicotinamide adenine dinucleotide (oxidized); NADH: Nicotinamide adenine dinucleotide (reduced)]

The workflow for the glycosidase plate-based assay is shown in Fig. 1a. IgG purified from human plasma sample is transferred to a 384 well plate as a set of three sub-samples (Supplementary fig. S1). The sub-samples without exoglycosidase treatment are used for quantification of IgG glycoprotein amounts by measuring absorbance at 280 nm. For the sub-samples treated with only the galactosidase, the exposed galactose residues (non-sialylated residues) are released from the *N*-glycans and will be referred to here as terminal galactosylation (Fig. 1b). However, for the sub-samples treated with the combination of the galactosidase and a sialidase, all galactose residues on the *N*-glycan are released and will be referred to here as total galactosylation (Fig. 1b). After the exoglycosidase treatments, the released galactose monosaccharides are utilized in an enzymatic redox reaction which forms the fluorometric detection mechanism of the assay (Fig. 2). This fluorescence signal is used for calculating the molar amounts of terminal galactosylation and total galactosylation, and their difference is equivalent to the sialylation on the *N*-glycans (Fig. 1b). However, these measurements are cumulative of the abundance of galactosylation and sialylation on the IgG glycoproteins as well as the amount of IgG glycoprotein in samples. Hence, in-order to express only the changes in glycosylation traits, the measurements have to be normalized to account for the differences in IgG amounts. This was done as either 1) an absolute quantification of moles of galactose or sialic acid per μg of IgG glycoprotein, when normalizing to the measured glycoprotein amounts in the sub-samples or as 2) a ratio of terminal galactosylation to total galactosylation, when normalizing to the glycan traits itself. The latter will be referred to here as galactosylation index.

The principle for the enzymatic redox reaction is shown in Fig. 2. After the exoglycosidase treatment of the IgG sub-samples, the released β-D-galactose monosaccharides are oxidized to D-galactonic acid by a β-galactose dehydrogenase which in turn reduces NAD^+^ to NADH. The NADH is then oxidised by a diaphorase which enables the reduction of resazurin to the fluorescent compound resorufin in molar proportions stoichiometric to the released galactose monosaccharides. The general disadvantage of such enzymatic assays is that their accuracy is often limited to the specificity/activity of the enzymes, which in this case are the galactosidase, sialidase and galactose dehydrogenase. However, since the assay functions on the combined activity of these enzymes, the cumulative effect of improved specificity to galactose quantification could be achieved. Additionally, the incorporation of a mutarotase into the redox reaction helps maintain a β conformer majority of the released galactose which favors the β-galactose dehydrogenase activity and hence assay kinetics.

For details pertaining to assay development and optimization, please see section “Supplementary Methods”.

### Assay performance

**Fig. 3:**
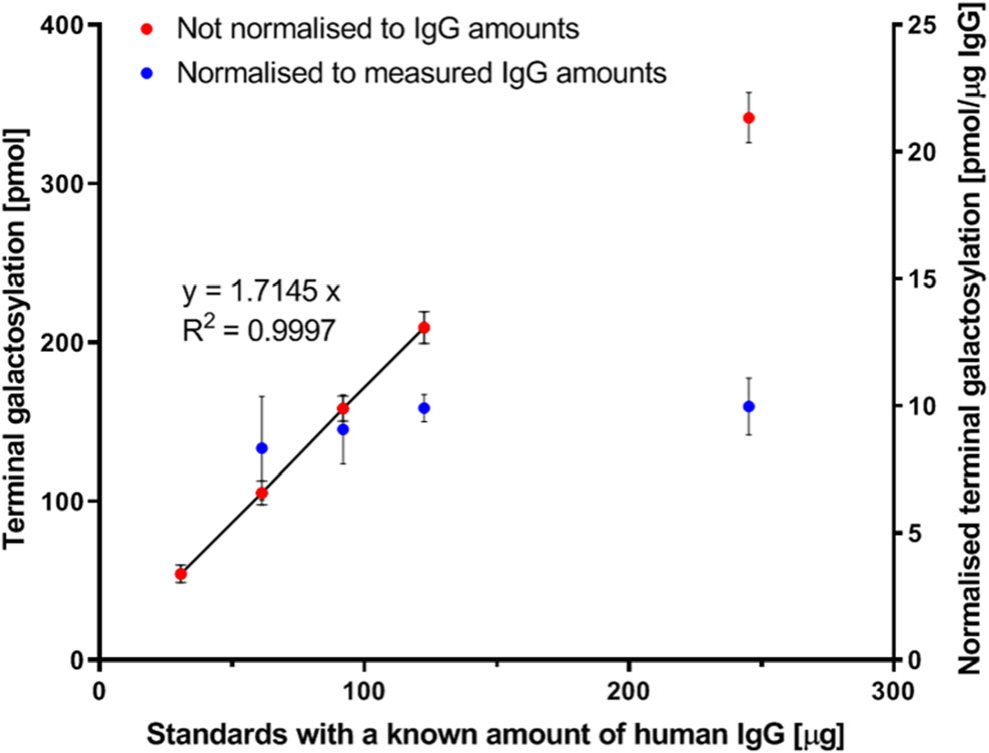
Linear range of quantification of the glycosidase plate-based assay for the quantification of terminal galactosylation on human IgG standards of known starting amounts. The terminal galactosylation are expressed as not normalized values (Red) and as values normalized to the measured IgG amounts in the sub-samples of assay (Blue). The line formula is representative of the linear range (solid trend linear) of the not normalized terminal galactosylation values. The normalized values for the lowest amount of IgG tested, 30 μg, was not calculable due to lack of measureable absorbance signals above background at 280 nm

The linear range of quantification for the assay, based on a galactose monosaccharides standard, is 42 pmols to 666 pmols (Supplementary fig. S2). However, for IgG glycoprotein standards, the linear range of quantification of not normalized terminal galactosylation is 54.1 ± 5.6 pmols to 209.3 ± 10.1 pmols of galactose which corresponds to 30 μg to 122 μg of IgG glycoprotein standards respectively (Fig. 3, Red). The loss of linearity of the not normalized values for amounts greater than 122 μg of IgG is potentially due to over saturation of the protein G resin that is used for affinity purification of the IgG, thus resulting in incomplete capture of IgG from the samples. This is demonstrated by normalizing the terminal galactosylation to the measured IgG amounts in the sub-samples of the assay which will account for the losses of IgG during the processing steps of the assay. Similar amounts of normalized terminal galactosylation were obtained for the standards with different starting amounts of IgG (Fig. 3, Blue). Furthermore, the normalized value of terminal galactosylation for the lowest amount of IgG tested, 30 μg, was not calculable due to lack of measureable absorbance signals above background at 280 nm (data not shown). Human serum contains 41 μg – 217 μg IgG per 10 μL of serum depending on the age and/or disease state of the patient [38, 39]. Our assay has been developed to perform within this range of IgG amounts (Fig. 3). However, to make a meaningful comparison between samples for the quantitated galactosylation and sialylation, these values will have to be normalized for the differences in IgG amounts between plasma samples as demonstrated in Fig. 3.

When cumulatively comparing the normalized values of galactosylation for samples with different amounts of IgG, the combined relative standard deviations (RSD) are much larger for the absolute quantification of galactosylation (pmols of galactose per μg IgG) on IgG compared to the galactosylation index (Supplementary fig. S3B). As mentioned above, the galactosylation index is the ratio of terminal galactosylation to total galactosylation in the IgG sample. On correlating terminal galactosylation to total galactosylation, a coefficient of 0.9947 was obtained (Supplementary fig. S4). However, for absolute quantification, on correlating the measured IgG glycoprotein amounts to terminal galactosylation or total galactosylation, lower coefficients of 0.9597 or 0.9652 were obtained, respectively (Supplementary fig. S5). The somewhat stronger correlations between terminal galactosylation and total galactosylation, makes the galactosylation index more robust at normalization of variations in IgG amounts, resulting in a lower combined RSD for the samples with different amounts of IgG glycoproteins (Supplementary fig. S3). The weaker correlation of the measured IgG glycoprotein amounts to terminal galactosylation or total galactosylation may be explained by the high variation in protein quantification using absorbance at 280 nm in the assay. These variations may be attributed to the 384 well plate, interferences in the sample and/or variation in path lengths at the meniscus of the viscous sample solution.

**Fig. 4:**
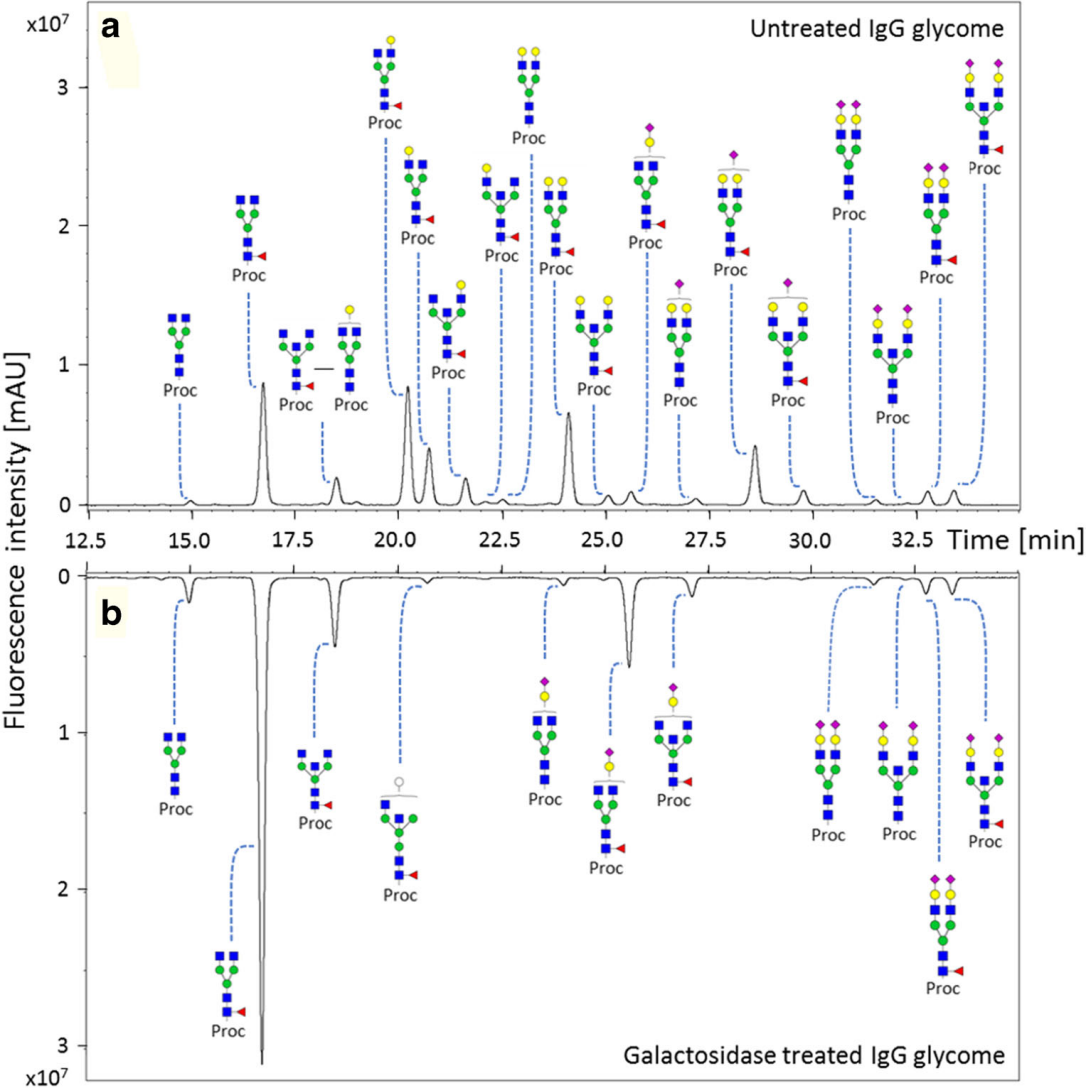
Confirmation of complete de-galactosylation of terminal galactose residues of *N*-glycans on IgG glycoproteins by the exoglycosidase treatment in the glycosidase plate-based assay. The released *N*-glycans from IgG glycoproteins (**a**) without galactosidase treatment was compared with (**b**) galactosidase treatment on a HILIC-FLD-MSn platform after labelling the reducing end with procainamide. For the confirmation of complete de-galactosylation and de-sialylation of *N*-glycans on IgG glycoproteins please see supplementary fig. S6. [Proc: procainamide; Blue square: *N*-acetylglucosamine, green circle: mannose, yellow circle: galactose, white circle: ambiguous hexose, red triangle: fucose, pink diamond: *N*-acetylneuraminic acid]

The exoglycosidase treatment of the IgG sub-samples in the assay are performed on intact IgG *N*-glycans (PNGaseF untreated). Hence there is a possibility that the conformation and intra-IgG molecular interactions [10, 40] of the *N*-glycans and/or the protein domains could restrict the activity of the exoglycosidases. This is especially important since the Fc *N*-glycans are largely located in the cavity formed by the Cγ2 and Cγ3 domains of the Fc homodimers and makes several interactions with the protein backbone [10]. However, in-order to contribute to the robustness of the assay, it is essential to ensure a complete de-sialylation and de-galactosylation of all *N*-glycans on IgG. To demonstrate this, the IgG sub-samples treated with and without exoglycosidases in the assay, were collected and their glycans released. The released glycans were labelled with procainamide and analyzed on a HILIC-FLD-MSn platform (Fig. 4). For the exoglycosidase untreated IgG sub-samples, 70.9% ±0.8% abundance of the glycome consist of glycans having one or more exposed terminal galactose residues (Supplementary Table S1). However, no such terminal galactosylated structures were detected after galactosidase treatment (Fig. 4b and Supplementary Table S2). Similarly, no detectable sialylated or galactosylated structures were observed after a combined sialidase and galactosidase treatment of IgG sub-samples, which was used for quantification of total galactosylation (Supplementary fig. S6 and Supplementary Table S3). Thus, the exoglycosidase treatment is not restricted to certain glycan structures or by the conformations and the molecular interactions of the glycan on the IgG molecule or by the IgG domains itself. The annotation list of the IgG glycans with and without exoglycosidase treatment are shown in Supplementary Table S4, S5 and S6.

**Fig. 5:**
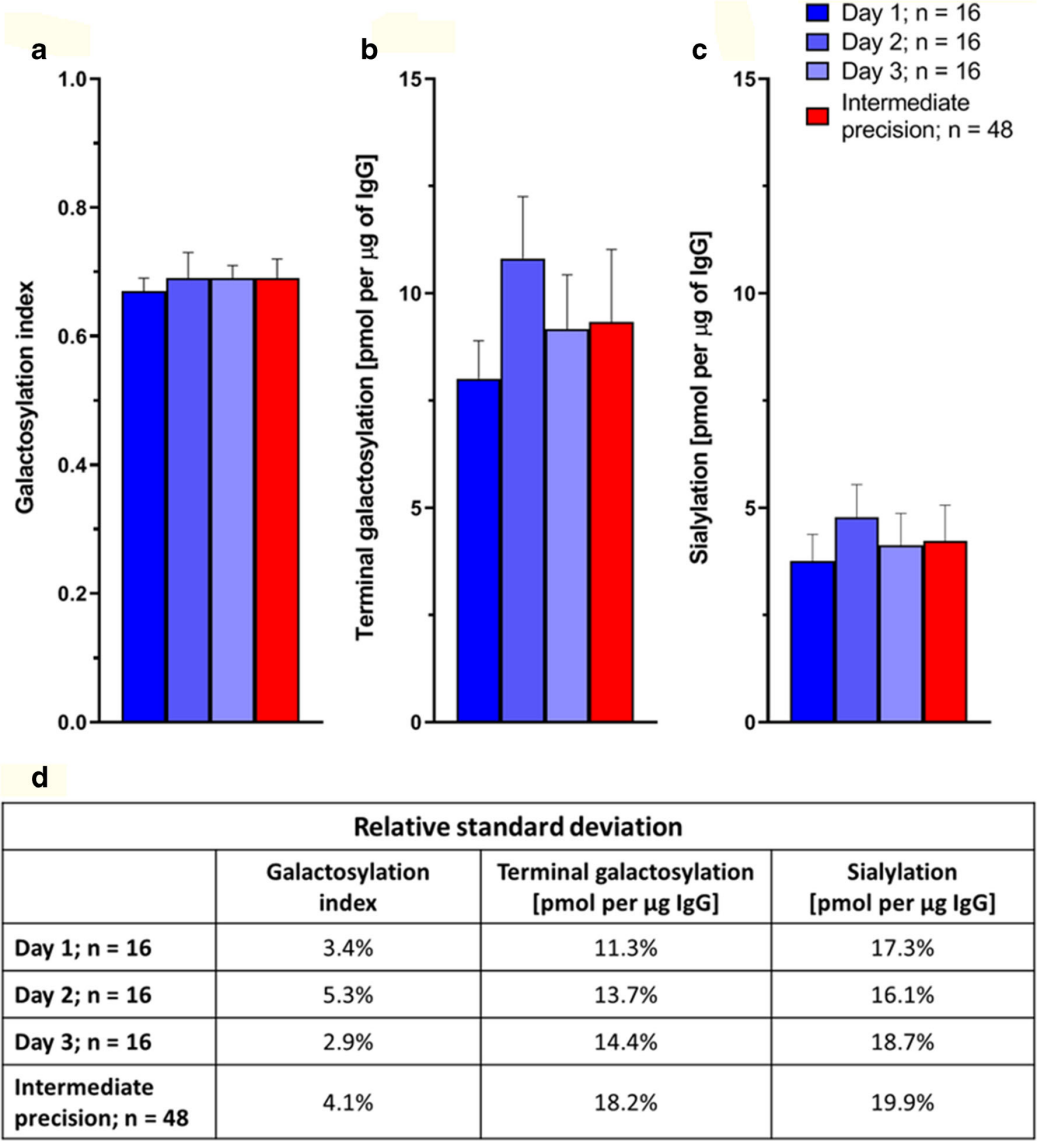
Intermediate precision of sample preparation and measurements for the glycosidase plate-based assay on IgG from pooled human plasma. The mean (**a**) galactosylation index, (**b**) terminal galactosylation [pmol] per μg IgG glycoprotein and (**c**) sialylation [pmol] per μg IgG glycoprotein are shown with error bars representing the standard deviation (*n* = 16). (**d**) The relative standard deviation are shown. [$Galactosylation\ index=\frac{\mathrm{Terminal}\ \mathrm{galactosylation}}{\mathrm{Total}\ \mathrm{galactosylation}}$]

Intermediate precision of sample preparation and measurement of the assay was assessed by performing three independent experiments on different days each with 16 replicates of IgG purified from pooled human plasma (Fig. 5). The galactosylation index was 0.69 with an RSD of 4.1% (Fig. 5a and d). This was similar to the galactosylation index calculated from procainamide labelled IgG glycans measured on a HILIC-FLD-MSn platform which was 0.729 with an RSD of 0.5% (Supplementary Table S7). These values signify that ~70% of all galactose residues on human IgG glycans are terminally exposed (terminal galactosylation) whilst the remaining 30% are capped by sialic acid residues. Alternatively, this can be stated as the amount of sialic acid residues are 30% of the total galactose residues. In a general comparison to some sandwich immuno/lectin-based glycomic assays, the intermediate precision was comparable [30], if not lower [29, 41]. Although the performance of such assays highly depends on the quantitated glycan traits, glycoprotein of interest, the matrix of the sample and/or the specificity of the lectins and antibodies used in assay. Furthermore, for our assay, the absolute quantification of terminal galactosylation was 9.3 pmol per μg IgG while the sialylation was 4.2 pmols per μg IgG, with RSDs of 18.2% and 19.9% respectively (Fig. 5b, c, d). These higher RSD can be explained again by likely variations in quantification of glycoprotein amounts using absorbance at 280 nm.

Both the absolute quantification of galactosylation or sialylation and the galactosylation index have their own advantages and disadvantages which may depend on the sample type and the purpose of the analysis. The low RSDs associated with the galactosylation index make it suitable for the comparison of clinically relevant samples where the biological relevant variations in galactosylation and sialylation could be limited. Moreover, it is a single value that gives the relative abundance of both galactosylation and sialylation, which on IgG glycans, are associated with pro-inflammatory and anti-inflammatory states respectively. Although the absolute quantification is associated with high RSDs, it has significance in the characterization and/or routine quality control analysis of certain sample types such as bio-pharmaceutical recombinant monoclonal antibodies. This is because it provides information on the number of galactose or sialic acid residues per molecule of the pure glycoprotein. Furthermore, the absolute quantification entails the level of agalactosylation on IgG while the galactosylation index omits such estimates. This reflects the major drawback of the galactosylation index since agalactosylation has also been associated with pro-inflammatory states.

### Comparison of glycosidase plate-based assay and HILIC-FLD-MSn for analysis of patient galactosylation index

**Fig. 6:**
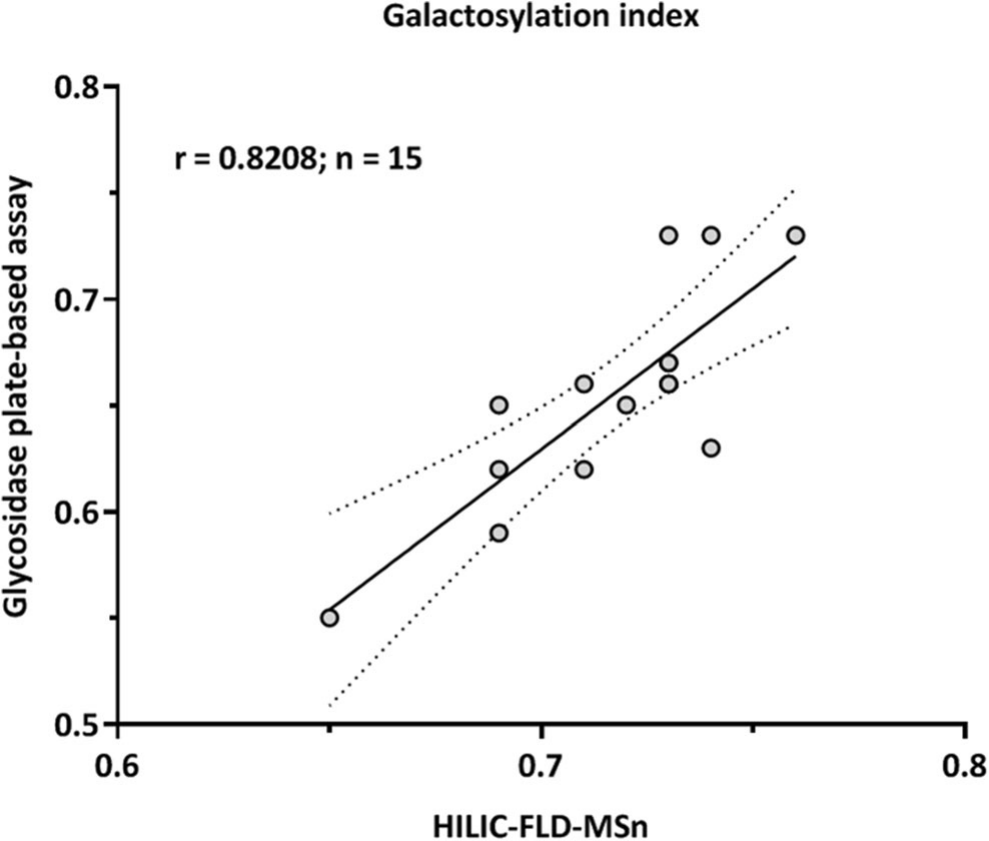
Correlation of the galactosylation index of IgG from human serum measured by the glycosidase plate-based assay, and by HILIC-FLD-MSn analysis of procainamide labelled released N-glycans. The human serum sample (*n* = 15) were obtained from an IBD cohort. A Pearson’s r correlation coefficient of 0.8208 was calculated with a *p* value of 0.0002. The trendline drawn is a linear regression of the data points with the error lines as 95% confidence intervals

The purpose of this research is to introduce the glycosidase plate-based assay as a prototype method of an initial screening assay for IgG galactosylation in a patient population. Following this, patients which may have a likely change in galactosylation, sialylation or galactosylation index can selectively be used for a further in-depth IgG glycome analysis such as on a HILIC-FLD-MSn platform. HILIC-FLD-MSn is a well-established technique that is widely used in industry for the identification and quantification of glycosylation changes in IgG. In order to establish the potential of the glycosidase plate-based assay to be used as an initial screening assay, we wanted to quantitatively compare the galactosylation index of this this technique with that of the HILIC-FLD-MSn method. We chose the galactosylation index as the appropriate value for making our comparison as it can be precisely calculated from the glycan profiles obtained by HILIC-FLD-MSn while neglecting the differences in IgG glycoprotein amounts. The analysis was performed on 15 patient plasma samples suspected of having IBD. The correlation of the methods resulted in a Pearson’s r correlation coefficient of 0.8208 with *p* value 0.0002 (Fig. 6 and Supplementary Table S8). Although the assay demonstrated a good correlation to the HILIC-FLD-MSn method, the lack of a stronger correlation cannot simply be explained by the measurement variations of the assay as RSDs of <5% were obtained for IgG from a pooled human plasma standard (Fig. 4). However presence of a quantification biasness between the methods could be explained by the O-glycans on IgG3 [42] and/or the presence of co-purified or contaminating proteins having O-glycosylation after the protein G purification of IgG from plasma. These undesirable glycoproteins could contribute to the quantified terminal and total galactosylation in the glycosidase plate-based assay and skew the galactosylation index of the patient samples. Notably the activity of the exoglycosidases used in the assay has not been tested on intact O-glycans of proteins. Additionally, the co-elution of multiple glycans under a fluorescence peak on the HILIC-FLD-MSn platform may also skew the calculated galactosylation index relative to the glycosidase plate-based assay.

The advantages of the glycosidase plate-based assay over HILIC-FLD-MSn are that it does not require extensive investments in high-end instrumentation techniques, software packages or technical skills. The only analytical instrument required is a fluorescence and absorbance microplate reader. We have demonstrated the assay as a semi-automated method on a robotic platform, but it can also easily be performed manually with the appropriate multichannel pipette. Once the samples have been processed in the assay, the measurement can be performed very rapidly in less than 5 min for measuring 96 samples, as opposed to several days on a HILIC-FLD-MSn platform. Furthermore, data processing is simple and quick as it simply provides the change in galactosylation and sialylation on IgG in the form of measured fluorescence intensities. However, a disadvantage of the assay is the lack of detailed information on glycosylation changes. It does not relay any information on the extent of galactosylation or sialylation on the different glycan structures such as bisecting or fucosylated structures. Hence, we do recognize the analytical benefits of the HILIC-FLD-MSn technique. However, the purpose of this assay can be two fold. As a simplistic initial screening assay to identify the samples that require further in-depth IgG glycomic analysis. Alternatively, where an established glycan related biomarker has already been demonstrated using high-end instrumentation, this assay could be used as a simpler analytical solution to focus on the ‘diagnostic’ glycan feature.

## Conclusion

We developed a prototype for a simple glycosidase plate-based assay for the quantification of galactosylation and sialylation traits on intact human IgG glycoproteins. The measured galactosylation and sialylation were normalised for the natural variation of IgG amounts in plasma by using the measured IgG glycoprotein amounts (absolute quantification) or by using the ratio of the glycan traits itself (galactosylation index). The latter has the advantage of lower intermediate RSDs (<5%) which has importance in comparing clinical relevant samples, whilst the former, which have much higher RSDs (~20%), has importance in characterization of glycoproteins such as biopharmaceuticals, since it provides information of the moles of galactose or sialic acid per mole of glycoprotein. Finally, we demonstrated the performance of the assay on clinically relevant human patient serum samples and obtained a Pearson’s correlation coefficient of 0.8208 (*p* = 0.0002) on comparison of the measured galactosylation index to an industrially established HILIC-FLD-MSn platform. The next step would be to demonstrate the assay on a much large clinically relevant patient cohort. Furthermore, it will be interesting to investigate the versatility of this method for the quantification of galactosylation and sialylation on other immunoglobulins such as IgA and IgM and/or also non-human mammalian immunoglobulins that may contain different patterns of galactosylation and sialylation. Finally, it must be stated that although the optimized assay were performed with non-commercial exoglycosidases, there is no reason for the assay not be performed with other such exoglycosidases which may or may not be commercially sourced.

## Electronic supplementary material

ESM 1 (DOCX 946 kb)

ESM 2 (XLSX 259 kb)

## Data Availability

The datasets generated for this study are available on request to the corresponding author.
