# Supplementary material for: A novel glycosidase plate-based assay for the quantification of galactosylation and sialylation on human IgG
Source: Glycoconj J. 2020 Oct 16;37(6):691–702. doi: 10.1007/s10719-020-09953-9 (PMC7679266; doi:10.1007/s10719-020-09953-9)
Supplement: Supplementary file 1 — (DOCX 946 kb) [file 10719_2020_9953_MOESM1_ESM.docx]

A novel glycosidase plate-based assay for the quantification of galactosylation and sialylation on human IgG.

Osmond D. Rebello^1, 2,*^, Richard A. Gardner^1^, Paulina A. Urbanowicz^1^, David N. Bolam^3^, Lucy I. Crouch^4^, David Falck^2^, Daniel I. R. Spencer^1^

^1^Ludger Ltd, Culham Science Centre, Abingdon, United Kingdom

^2^Center for Proteomics and Metabolomics, Leiden University Medical Center, Leiden, Netherlands

^3^Biosciences Institute, Faculty of Medical Sciences, Newcastle University, Newcastle upon Tyne, UK.

^4^Institute of Microbiology and Infection, School of Biosciences, University of Birmingham, Birmingham B15 2TT.

*****Corresponding: O.D.Rebello@lumc.nl, Daniel.Spencer@ludger.com

**Supplementary Methods**

**Assay development and optimisation**

Experiments involving the development of the assay was performed with samples of human gamma globulin (Sigma, United Kingdom) which is a natural enriched source for IgG glycoproteins. Furthermore, some of these assay development experiments were performed with a commercially available β-galactosidase (E-BG07, QABio, USA) and α-galactosidase (E-AG02, QABio) (data not shown). For these development experiments, IgG purification was not performed. Heat denatured and native IgG glycoproteins were treated with and without PNGaseF during the overnight exoglycosidase treatment, so as to assist whether *N*-glycan release would aid the exoglycosidase activity. Heat denatured glycoprotein samples resulted in very low galactose quantification as compared to the native samples, regardless of the presence of PNGaseF (Supplementary figure S7). The limited exoglycosidase activity observed for the heat denatured samples might be explained by the lack of non-ionic detergents such as NP-40 that is known to have a protective role against protein aggregation which can be promoted by denatured proteins. The use of non-ionic detergents was not investigated here as there were foreseeable issues with liquid handling in the assay such as excess bubble formation. Besides, the exoglycosidase treatment was also determined to reach completion with the native IgG glycoproteins (Figure 4 and Supplementary figure S6). Next, in order to reduce the variation of the quantification, we firstly aimed at improving the signal response of the fluorometric enzymatic redox reaction which forms the detection mechanism of the assay and then the parameters in the fluorometric microplate reader (see subsection “Preparation of enzymatic redox reaction and fluorometric measurement of assay”). A maximum fluorescence intensity for the samples were observed with the use of 10 – 25 mM NAD+ in the reagent mix (Supplementary Figure S8). Lower concentrations of NAD+ may result in its limitation for the completion of the redox reaction while higher concentrations might result in substrate inhibition of the diaphorase and hence a reduce fluorescence intensity for the assay. Furthermore, a maximum fluorescence intensity for the samples were observed with the use of 200 – 1000 μM resazurin in the reagent mix (Supplementary Figure S9). Once again, lower concentration of resazurin may result in its limitation for the completion of the redox reaction while higher concentration might result in fluorescence quenching of the resorfurin and/or substrate inhibition of the diaphorase. Hence all further experiments were performed with 25 mM NAD+ and 1000uM resazurin. Furthermore, the enzyme mix prepared with 4U/mL diapharose and 1 μL of the enzyme solution of galactosidase dehydrogenase and galactose mutarotase (K-Arga, Megazyme), was found to be optimal for the redox reaction (data not shown). For these optimal conditions, this enzymatic redox reaction reaches completion at 60 minutes and is stable until 300 minutes, although further time points were not investigated (Supplementary Figure S10). The redox reaction of all further experiments were measured at 180 minutes so as to facilitate the robustness of the detection mechanism.

For the optimised conditions of the assay, the use of IgG glycoproteins purified from human plasma were incorporated as samples for the assay. This involved a minor optimisation of the IgG purification method [1] as it was important to ensure a robust neutralisation of the acidic eluent containing the IgG glycoprotein. An incomplete neutralisation will result in acidification of the exoglycosidase reactions and hence decrease activity of the exoglycosidases. 10 mM formic acid was finalised as eluent for the purified IgG from protein G beads as it is allowed for a complete neutralisation by the buffer (125mM SPBS, pH 6) of the exoglycosidase mix (data not shown).

Galactosylated glycans makes up 80.3% ±0.9% abundance of the human IgG glycome (Supplementary table S1). 70.9% ±0.8% abundance of this glycome compromises of glycans with one or more terminal galactose residues, while 24.3% ±1.3% of its abundance compromise of sialylated glycans (Supplementary table S1). Hence it was important to ensure a robust and complete degalactosylation and desialylation of the glycans on native IgG glycoproteins purified from human plasma. A complete degalactosylation of terminal galactose residues was achieved with 0.1 μM galactosidase (Supplementary figure S11). However to facilitate a robust and complete degalactosylation, a 20 fold excess of 2 μM galactosidase was used chosen. Additionally, for the purpose of quantification of total galactosylation, and hence sialylation, a complete desialylation of these glycans was also necessary (Supplementary figure S12). All further experiments were performed with 2 μM galactosidase for quantification of terminal galactosylation, and with a combination of 2 μM galactosidase and 5 μM sialidase for quantification of total galactosylation. Under these exoglycosidase conditions, complete degalactosylation and desialylation was confirmed by glycan analysis on a HILIC-FLD-MSn platform (Figure 4 and Supplementary figure S6). Additionally, the galactosidase Bt0461 is specific to β(1-4) galactose linkage (Supplementary figure S13 and S14), and is not restricted by the linkage variants of the penultimate GlcNAc residues on the *N*-glycan (Supplementary figure S15). For experiments analysing the linkage specificity of the galactosidase Bt0461, glycan standards were treated with galactosidase Bt0461 and with a commercially available β-galactosidase (E-BG07, QABio, USA) and a α-galactosidase (E-AG02, QABio). These treated and untreated glycans were then procainamide labelled and analysed on a HILIC-FLD-MSn platform as mentioned in subsection *Hydrophilic interaction liquid chromatography analysis of IgG N-glycans.*

**Supplementary figures**


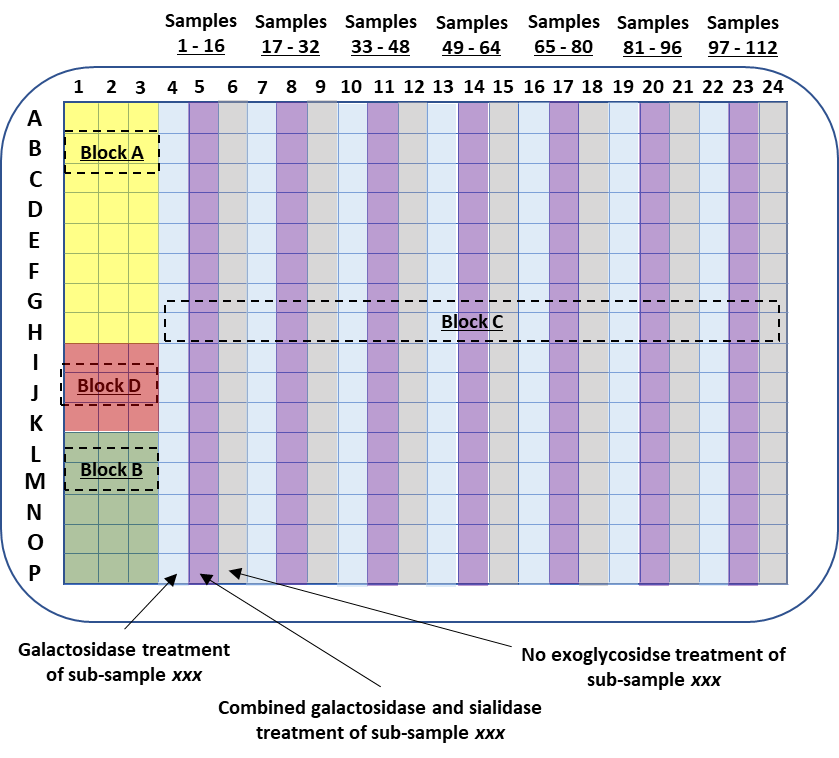


**Supplementary figure S1**. General map of sample arrangement for the 384 well plate used in the glycosidase plate-based assay. Block A (Yellow wells) are allocated for the galactose monosaccharide standard solutions which provides the fluorescence standard curve for galactose quantification. Block B (Green wells) are allocated for the IgG glycoprotein standards which provides the absorbance standard curve for IgG glycoprotein quantification of the samples. Block C are allocated for the sample of IgG glycoprotein purified from human plasma. Each sample is divided and processed as three sub-samples that receive 1) galactosidase treatment (Blue wells), 2) a combined galactosidase and sialidase treatment (Purple wells) and 3) no exoglycosidase treatment (Grey wells). Block D (Red wells) are allocated for checking the presence of interferences or background coming from the galactosidase mix, the galactosidase and sialidase mix and from the buffer (125 mM SPBS) which that is used in the exoglycosidase treatment.


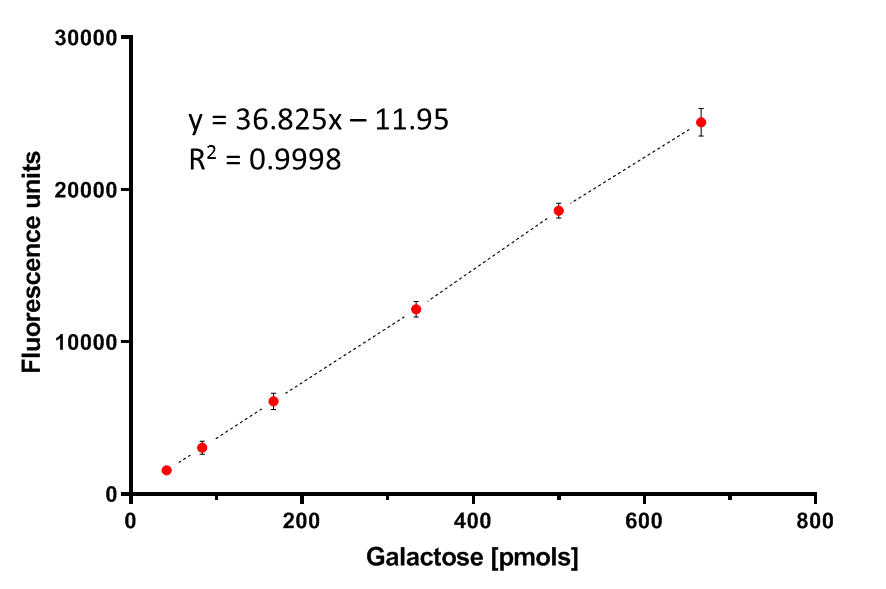


**Supplementary figure S2**. Linear range of quantification for the glycosidase plate-based assay. The assay was performed with galactose monosaccharide standard ranging in amounts from 8 pmols to 666 pmols. The line formula is shown for the linear range of the curve which is 42 pmols to 666 pmols. The error bars represent the standard deviation of the mean (n = 3).


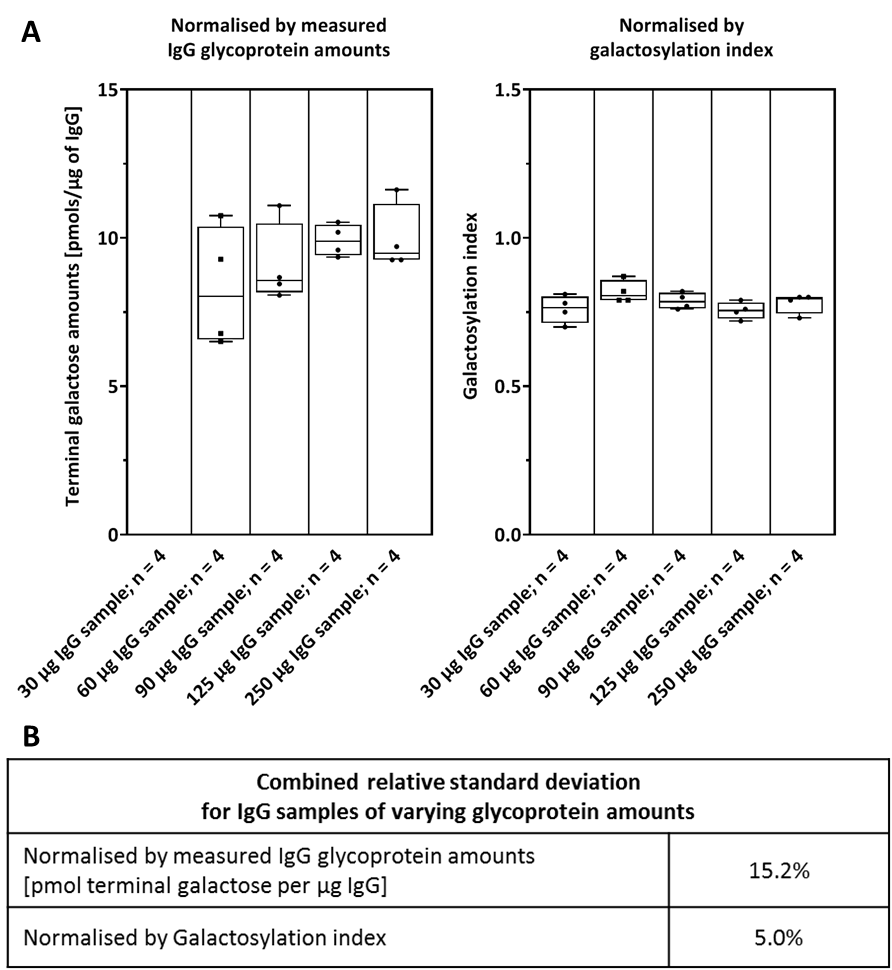


**Supplementary figure S3**. Comparison of normalisation potential for galactosylation on IgG molecules measured using the glycosidase plate-based assay. The assay was performed with samples (n = 4) containing 30 µg, 60 µg, 90 µg, 125 µg and 250 µg IgG glycoproteins. The measured galactosylation is normalised for the differences in IgG amounts between samples by **(A, left)** the measured glycoprotein amounts determined from absorbance at 280 nm and by **(A, right)** the galactosylation index which is the ratio of terminal galactosylation to total galactosylation. Protein quantification of the 30 µg IgG sample was not possible as the absorbance at 280 nm was below the detection limit. **(B)** The combined relative standard deviation of the normalised values for all the samples containing varying amounts of IgG glycoprotein are shown in the table.


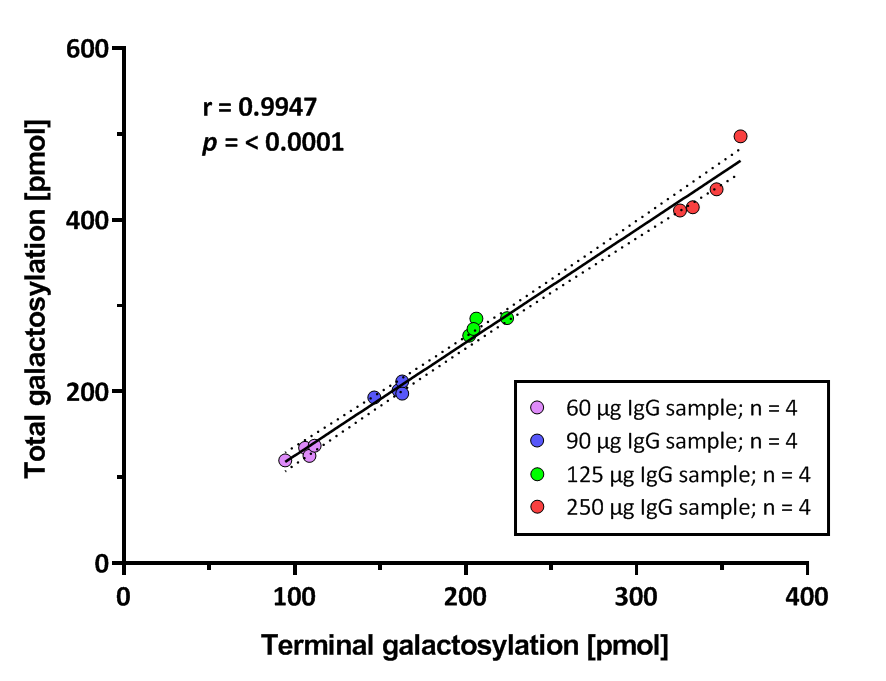


**Supplementary figure S4**. Correlation plot of terminal galactosylation to total galactosylation, for samples containing varying amounts of IgG glycoproteins in the glycosidase plate-based assay. The Pearson coefficient (r) and *p* values (α = 0.05) are shown in the plots.


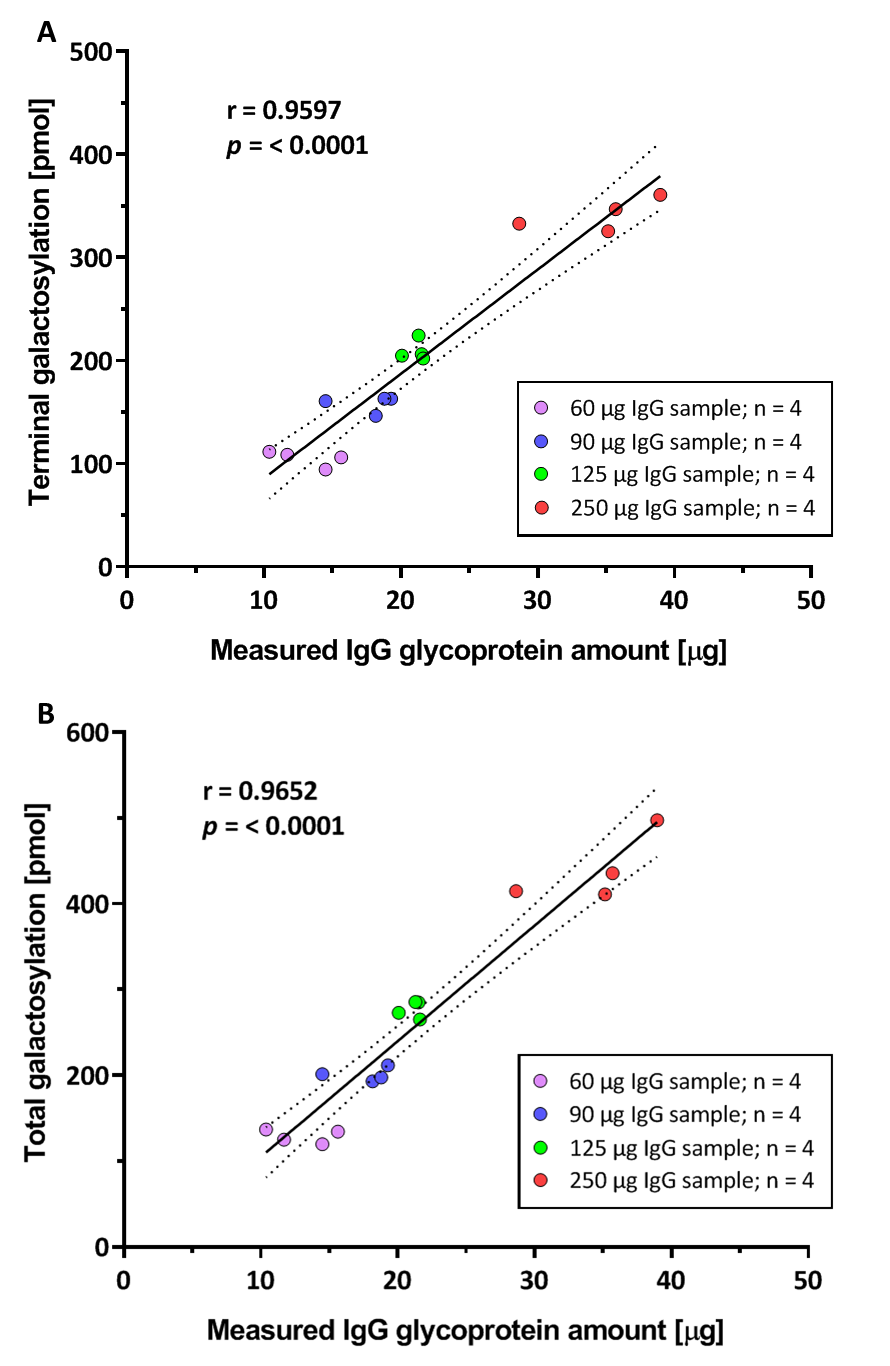


**Supplementary figure S5**. Correlation plots of the measured glycoprotein amounts (absorbance 280 nm) of purified IgG to **(A)** terminal galactosylation and **(B)** total galactosylation, for samples containing varying amounts of IgG glycoproteins in the glycosidase plate-based assay. The Pearson’s r correlation coefficients and *p* values (α = 0.05) are shown in the plots.


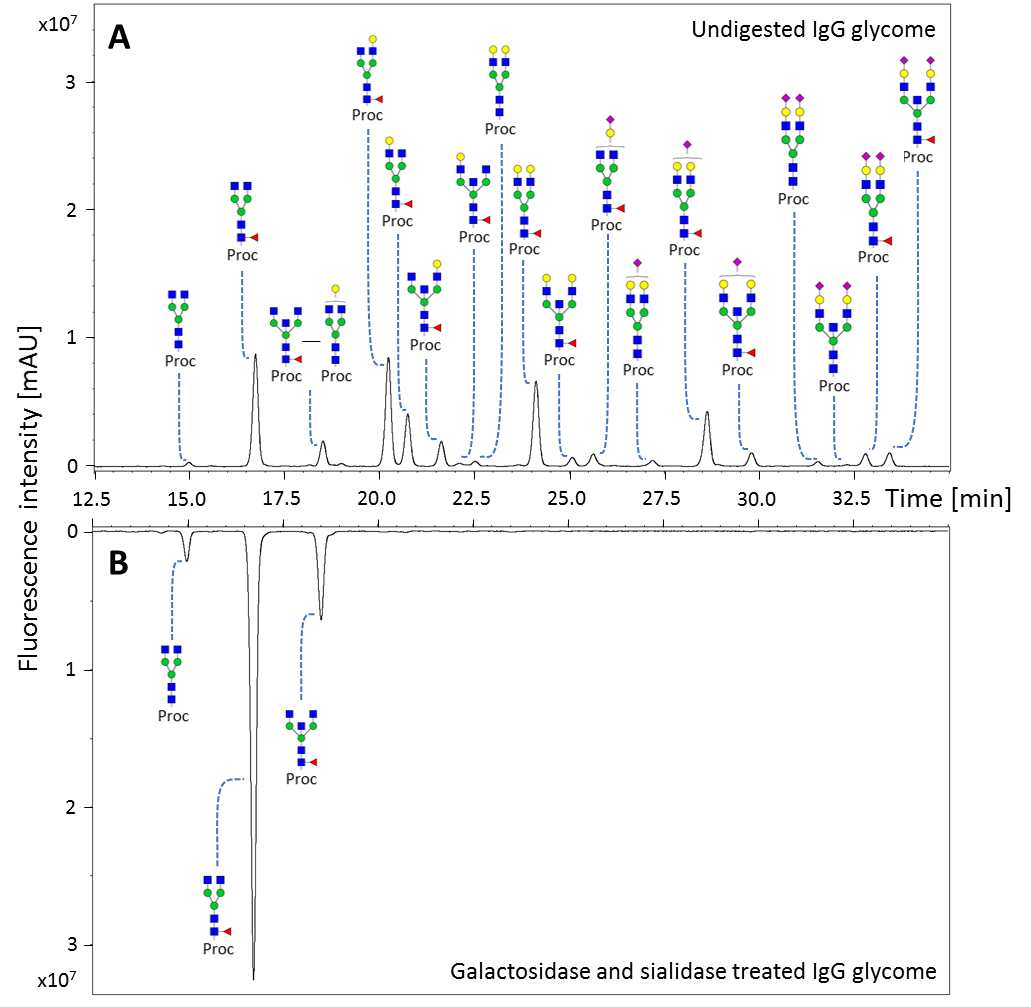


**Supplementary figure S6.** Confirmation of complete de-sialylation and de-galactosylation of *N*-glycans on IgG glycoproteins by the exoglycosidase treatment in the glycosidase plate-based assay. The released *N*-glycans from IgG glycoproteins (**A**) without exoglycosidase treatment was compared with (**B**) sialidase and galactosidase treatment on a HILIC-FLD-MSn platform after labelling the reducing end with procainamide. [Proc : procainamide; Blue square: *N*-acetylglucosamine, green circle: mannose, yellow circle: galactose, red triangle: fucose, pink diamond: *N*-acetylneuraminic acid].

**Supplementary figure S7**. Comparing the effects of IgG glycoprotein heat denaturation, with and without subsequent PNGaseF treatment in the glycosidase plate-based assay. 100 µg of IgG glycoproteins (source: human gamma globulin) was heat denatured at 95°C for 10 minutes. Both the heat denatured and not denatured glycoproteins where treated with and without PNGaseF in the overnight galactosidase treatment step of the glycosidase plate-based assay. The error bars represent the standard deviation of the mean (n = 3).


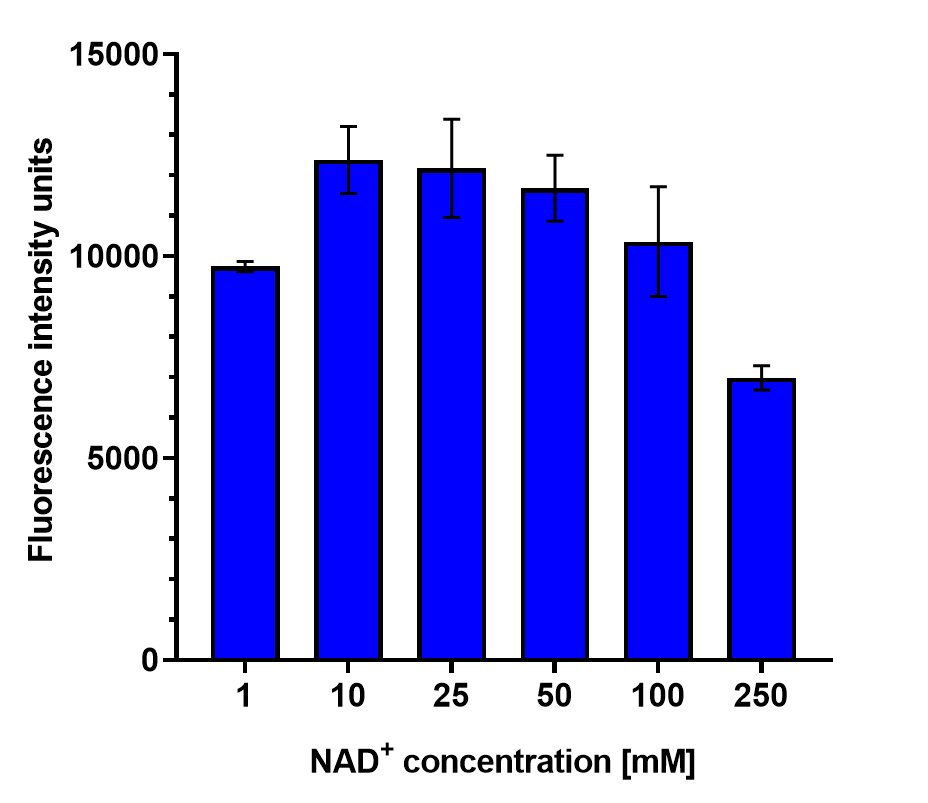


**Supplementary figure S8**. Comparing the effects of NAD+ concentration in the enzymatic redox reaction that forms the detection mechanism of the glycosidase plate-based assay. The reagent mix used in the redox reaction of the assay were prepared with 1 mM, 10 mM, 25 mM, 50 mM, 100 mM and 250 mM NAD+ stock solutions. The error bars present the standard deviation of the mean (n = 3).


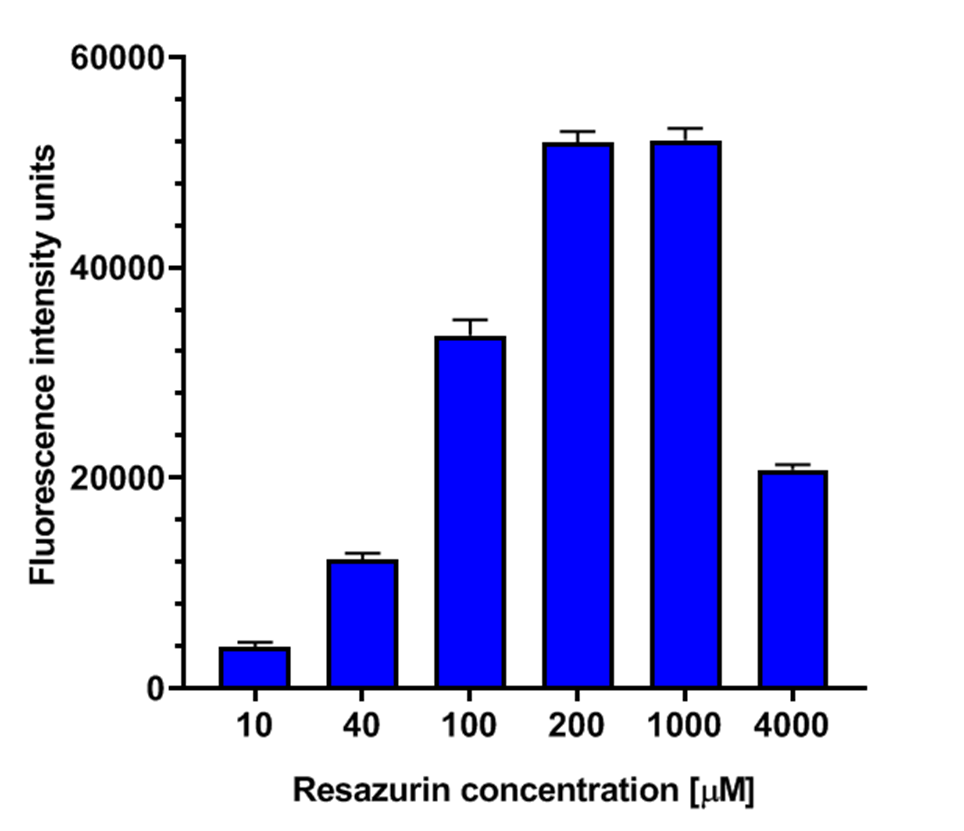


**Supplementary figure S9**. Comparing the effects of resazurin concentration in the enzymatic redox reaction that forms the detection mechanism of the glycosidase plate-based assay. The reagent mix used in the redox reaction of the assay were prepared with 10 µM, 40 µM, 100 µM, 200 µM, 1000 µM and 4000 µM resazurin stock solutions. The error bars present the standard deviation of the mean (n = 3).

**Supplementary figure S10**. Comparing different incubation times for the enzymatic redox reaction that forms the detection mechanism of the glycosidase plate-based assay. The fluorescence output of the redox reaction was measured at 60 minutes, 120 minutes, 180 minutes, 240 minutes and 300 minutes, during the incubation period. The error bars represent standard deviation of the mean (n = 3).


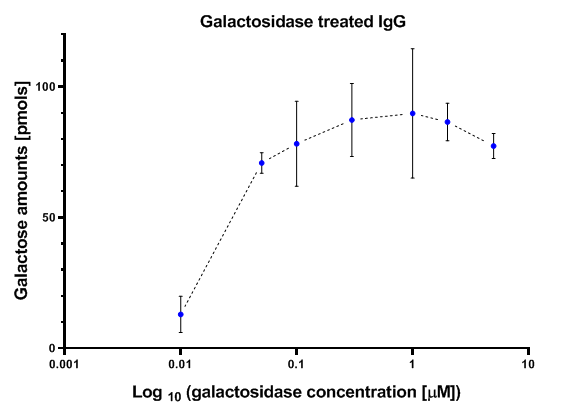


**Supplementary figure S11**. Residual terminal galactosylation on human IgG after incubation with varying amounts of Bt0461 β1,4-galactosidase. The exoglycosidase treatment step of IgG samples in the glycosidase plate-based assay was performed with 0.01 µM, 0.05 µM, 0.1 µM, 0.3 µM, 1 µM, 2 µM and 5 µM galactosidase. The error bars represent the standard deviation of the mean (n = 4).


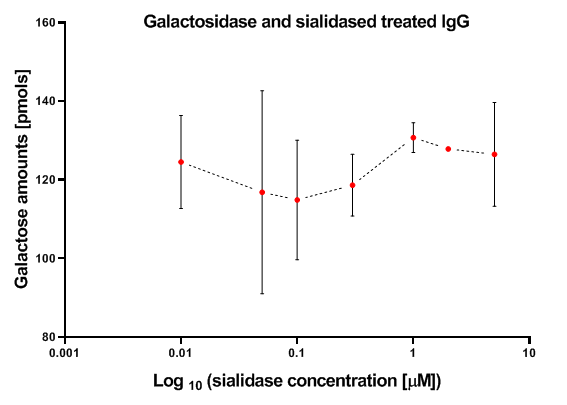


**Supplementary figure S12**. Residual total galactosylation on human IgG after incubation with varying amounts of sialidase along with 2 µM β-galactosidase. The exoglycosidase treatment step of IgG samples in the glycosidase plate-based assay was performed with 2 µM galactosidase along with either 0.01 µM, 0.05 µM, 0.1 µM, 0.3 µM, 1 µM, 2 µM and 5 µM of sialidase. The error bars represent the standard deviation of the mean (n = 4).


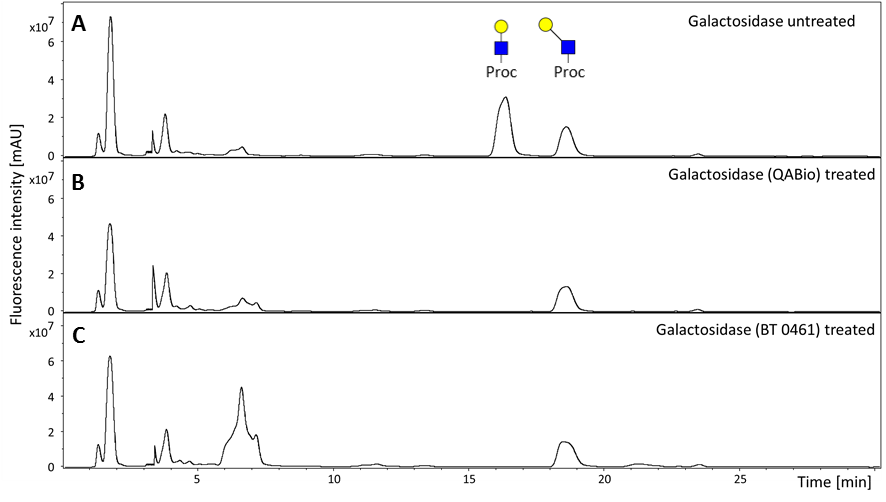


**Supplementary figure S13.** Confirming galactosidase specific to β(1-4) linked galactose residues. An (A) untreated near equal molar mixture of disaccharides, Gal β(1-4) GlcNAc and Gal β(1-3) GlcNAc, is compared to its treatment with (B) a commercially available galactosidase (E-BG07, QABio, USA) and (C) β-galactosidase Bt0461 which was used the glycosidase plate-based assay. The commercially available galactosidase (E-BG07; QABio) is specific to β(1-4) linked galactose residues. [Proc : procainamide; Blue square: N-acetylglucosamine, yellow circle: galactose (0° β1,4-linked; −45° β1,3-linked)].


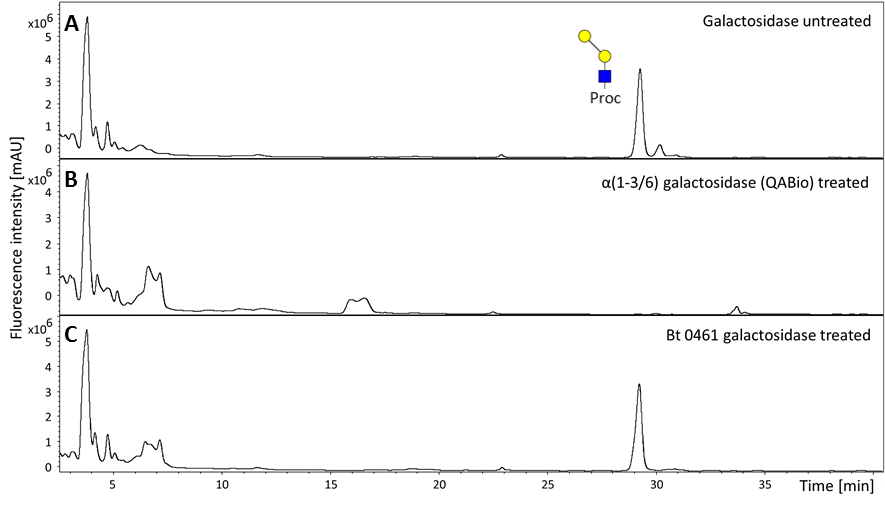


**Supplementary figure S14.** Confirming Bt0461 galactosidase does not cleave α(1-3) linked galactose residues. The (**A**) untreated trisaccharide Galα(1-3)Galβ(1-4)GlcNAc, is compared to its treatment with (**B**) a commercially available α-galactosidase (QABio, USA) and (**C**) β1,4-galactosidase Bt0461 which was used the glycosidase plate-based assay. The commercially available galactosidase (E-AG02) is specific to α(1-3/6) linked galactose residues. [Proc : procainamide; Blue square: *N*-acetylglucosamine, yellow circle: galactose (0° β1,4-linked; −45° α1,3-linked)].


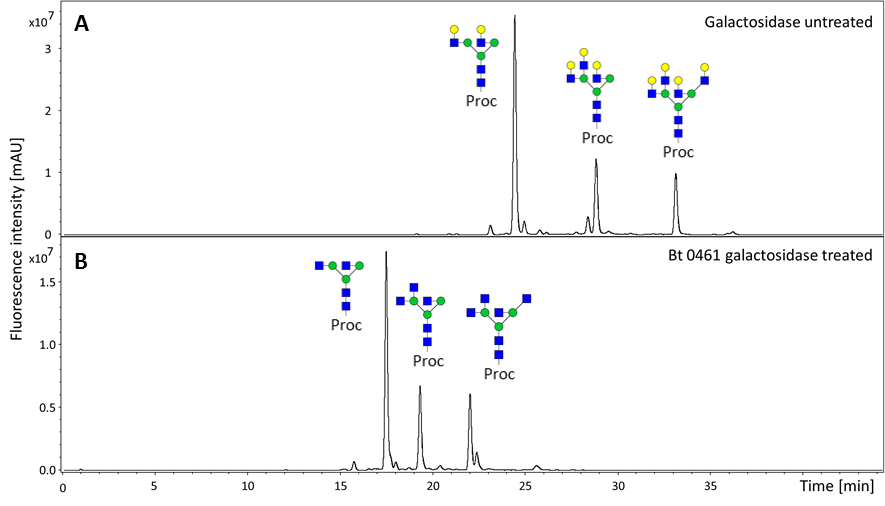


**Supplementary figure S15.** Confirming Bt0461 galactosidase specific to β(1-4) linked galactose residues on penultimate GlcNAc residues of varying linkages. An (**A**) untreated near equal molar mixture of N-glycans, A2G2, A3G3 and A4G4, is compared to its treatment with (**B**) galactosidase Bt0461 which was used the glycosidase plate-based assay. [Proc : procainamide; Blue square: *N*-acetylglucosamine (-90° β1,2-linked; 0° β1,4-linked; +45° β1,3-linked), green circle: mannose, yellow circle: galactose (0° β1,4-linked)].

1. Bondt, A., et al., *Immunoglobulin G (IgG) Fab glycosylation analysis using a new mass spectrometric high-throughput profiling method reveals pregnancy-associated changes.* Molecular & cellular proteomics : MCP, 2014. **13**(11): p. 3029-3039.
